# Circulating MicroRNAs as Biomarkers and Mediators of Cell–Cell Communication in Cancer

**DOI:** 10.3390/biomedicines3040270

**Published:** 2015-11-09

**Authors:** Molly A. Taylor

**Affiliations:** AstraZeneca, R&D Oncology iMed, Room 33F83/7 Mereside, Alderley Park, Macclesfield SK10 4TG, UK

**Keywords:** micron, circulating biomarker, cancer

## Abstract

The realization of personalized medicine for cancer will rely not only on the development of new therapies, but on biomarkers that direct these therapies to the right patient. MicroRNA expression profiles in the primary tumor have been shown to differ between cancer patients and healthy individuals, suggesting they might make useful biomarkers. However, examination of microRNA expression in the primary tumor requires an invasive biopsy procedure. More recently, microRNAs have been shown to be released from the primary tumor into the circulation where they can be utilized as non-invasive biomarkers to diagnose patients, predict prognosis, or indicate therapeutic response. This review provides an overview of the current use of circulating microRNAs as biomarkers as well as recent findings on their role in regulating cell signaling interactions in the tumor microenvironment.

## 1. Introduction

Cancer is a leading cause of death worldwide, with an estimated 14.1 million new cancer cases and 8.2 million deaths occurring in 2012 worldwide [1]. Despite these grim statistics, the development of targeted therapies has helped to decrease the annual cancer death rate by about 20% over the past twenty years [2]. These targeted therapies are designed to inhibit specific cellular signaling defects that occur in a subset of patients. For instance, trastuzamab (Herceptin; Roche/Genentech, San Francisco, CA, USA), is used to treat breast cancer patients with HER2 (ERBB2) amplifications, imatinib (Gleevec; Novartis, Basil, Switzerland), is used to treat chronic myeloid leukemia only in patients that have a breakpoint cluster region, Abelson (BCR-ABL) fusion gene, and gefitinib (Iressa; AstraZeneca, Alderley Park, UK) works well in patients that have an activating mutation in the epidermal growth factor receptor (EGFR) receptor [3]. As these examples illustrate, targeted therapies require the selection of a subset of patients that have a specific mutation or other disease driver. As such, development of new targeted therapies will require the parallel identification of new biomarkers to help pair the right therapy with the right patient. Indeed, biomarkers are increasingly used to diagnose patients (diagnostic biomarkers), predict survival (prognostic biomarkers), or measure therapeutic response. While advances in genomics and molecular biology have led to the identification of novel biomarkers and diagnostic gene expression signatures [4,5,6], there is a substantial need for additional blood-based biomarkers to circumvent the need for invasive biopsy procedures.

MicroRNAs are an evolutionarily conserved class of small non-coding RNAs that repress target gene expression at the post-transcriptional level through binding to the 3’UTR of the target mRNA. The first microRNAs discovered were lin-4 and let-7, which were identified to be translational repressors of mRNAs that encode proteins of the heterochronic developmental timing pathway in *C. elegans* [7]. After these discoveries, intense research efforts have revealed that microRNAs play a major role in cellular development, differentiation, disease pathology and tumor progression. Moreover, microRNAs have been found at high levels in the circulation, where recent evidence suggests they may carry out cell signaling functions. Additionally, these circulating microRNAs have great potential for use as non-invasive biomarkers. This review will highlight recent findings on the use of microRNAs as biomarkers and discuss novel findings on their role in cell–cell communication and is not meant as a comprehensive overview of all circulating microRNA biomarker studies. Readers desiring a more comprehensive overview are directed to several recent reviews [8,9,10].

## 2. MicroRNA Biogenesis and Function

MicroRNAs are first transcribed in the nucleus, usually by RNA Pol II, into a 70–100 nucleotide hairpin primary microRNA transcript (pri-miRNA), which is then cleaved by the Drosha complex to produce a precursor microRNA (pre-miRNA). This pre-miRNA is then exported from the nucleus by Exportin 5, where it is cleaved by the Dicer complex, generating a short RNA duplex. One strand of this duplex is selected for incorporation into the RNA-induced silencing complex (RISC) together with Argonaute (Ago2), which guides the complex to its target mRNA [11]. Base pairing between a microRNA and the 3′UTR of its mRNA target is mainly determined by the 6 to 8-nucleotide sequence at the 5′ end of the microRNA, termed the “seed” sequence. The extent of sequence complementarity between the 5′-seed region of the loaded microRNA with that of its target mRNA 3′UTR dictates the mechanism of mRNA silencing [11]. Perfect base-pairing homology usually leads to mRNA cleavage and degradation, while imperfect base pairing leads to repression of protein translation. A single microRNA is capable of targeting numerous mRNAs. Recent evidence indicates that microRNAs can also bind to other regions in a target mRNA and that some microRNAs may function in a “non-canonical” manner to increase translation of specific mRNAs through recruitment of protein complexes [12]. Importantly, misexpression of a single microRNA can disrupt the expression of hundreds of proteins, thereby promoting tumor progression [13].

## 3. MicroRNAs in Cancer

Genome-wide microRNA profiling studies have shown that effectively all cancers exhibit unique microRNA expression profiles [14,15]. Additionally, microRNAs play active roles in regulating virtually all of the physiological processes that lead to tumor development, progression, and metastasis [13]. MicroRNAs have been credited with the ability to be both potent tumor suppressors in normal cells and to be dynamic tumor promoters in developing and progressing carcinomas. For example, miR-17–92, one of the most well characterized oncomiRs, has been shown to be upregulated in a variety of human tumors [16], where it exerts anti-apoptotic effects by downregulating Bim and PTEN tumor suppressors [17,18]. Similarly, miR-21 and miR-155 are commonly upregulated across numerous cancer types and have been shown to promote tumor growth and metastasis in numerous contexts [13]. In contrast, let-7, miR-15a, miR-16-1, and miR-34a act as a potent tumor suppressor by inducing apoptosis, cell cycle arrest, and senescence by downregulating a number of oncogenic mRNA targets (Bcl2, cyclin D1, CDKs 4 and 6, c-Myc, MET, N-Myc, and SIRT1) [13]. Additionally, other microRNAs, such as miR-181a can play the role of both oncogene and tumor suppressor depending on the cellular context in which they are expressed [19]. The ability of microRNAs to target multiple genes and modulate numerous signaling pathways has created considerable interest in their potential clinical utility as biomarkers.

## 4. MicroRNAs as Biomarkers

In addition to the central role that microRNAs play in modulating target gene expression, microRNAs represent promising biomarkers. Examination of microRNA expression profiles in primary tumors indicates that microRNA profiles reflect developmental lineage and differentiation state and that microRNA profiles are more accurate in classifying tumors of unknown origin than previously used mRNA profiles [14]. Moreover, microRNA signatures in primary tumors have been observed to possess more predictive power than their larger and more extensive mRNA signature counterparts [20]. More recently, microRNAs have been shown to be released from the primary tumor into the circulation. These cell-free microRNAs can be detected in virtually all body fluids including the whole blood, serum, plasma, urine, and saliva and have been shown to be remarkably stable under harsh conditions and able to survive high temperatures, extreme pH, and RNase activity [21]. The stability of microRNAs in biofluids can be attributed to the fact that they are shielded from RNase degradation by complexing with Ago proteins, association with high-density-lipoprotein (HDL), and packaging into exosomes [22]. Given (*i*) their frequent dysregulation in cancer, (*ii*) their tissue specific expression patterns and (*iii*) their high stability in body fluids, circulating microRNAs represent ideal candidates for non-invasive biomarkers to diagnose disease, predict prognosis, and measure therapeutic response.

### 4.1. Circulating MicroRNAs as Diagnostic Biomarkers

Often, diagnosis of cancer relies heavily on imaging techniques, such as CT scans to diagnose lung cancer, mammograms to diagnose breast cancer, and ultrasounds to diagnose ovarian cancer [23]. These technologies often lead to false-positives, necessitating an invasive biopsy procedure to confirm results. Additionally, imaging techniques are often unable to distinguish between benign and malignant lesions. Although there are several circulating protein biomarkers currently in use to diagnose cancer, such as CA-125 for diagnosis of ovarian cancer, CA19-9 for pancreatic cancer, and prostate specific antigen (PSA) for prostate cancer, these traditional biomarkers are limited by low sensitivity and the inability to distinguish between aggressive and indolent forms of disease [23]. Circulating microRNAs may represent a new class of biomarkers to improve upon current diagnostic techniques. For instance, expression of 10 microRNAs (miR-20a, miR-24, miR-25, miR-145, miR-152, miR-199-5p, miR-221, miR-222, miR-223, and miR-320) in the circulation was shown to distinguish non-small cell lung cancer (NSCLC) patients from healthy controls. Moreover, this signature could accurately classify serum samples that were collected up to 33 months ahead of diagnosis [24]. Similarly, increased expression of miR-10b, miR-155, and miR-195 along with a decrease in expression of miR-34a has been observed in the circulation of breast cancer patients over controls [25]. In prostate cancer, circulating levels of miR-141 along with 15 other microRNAs (miR-16, miR-92a, miR-92b, miR-103, miR-107, miR-197, miR-34b, miR-328, miR-485-3p, miR-486-5p, miR-574-3p, miR-636, miR-640, miR-766, and miR-885-5p) have been found to be higher in patients than in healthy controls [21,26]. In addition, circulating miR-221 has been shown to be higher in patients with ovarian cancer, melanoma, and lymphoma compared to healthy controls [27,28,29]. Taken together these studies suggest that microRNA biomarkers may be more sensitive than current circulating protein biomarkers, and than imaging techniques in cancer diagnosis (Table 1). However, more work is needed to determine which microRNAs (like miR-221) are commonly found in the circulation of patients with multiple types of cancer and which microRNAs may distinguish specific cancer types.

**Table 1:** MicroRNAs with potential as circulating biomarkers for diagnosis of cancer.

Diagnostic miR(s)	Disease Setting	Description	Reference(s)
miR-20a, miR-24, miR-25, miR-145, miR-152, miR-199-5p, miR-221, miR-222, miR-223, miR-320	Lung Cancer	10 microRNAs were found to have significantly different expression levels in NSCLC serum samples compared with the control serum samples.	[24]
miR-10b, miR-155, miR-195, miR34a	Breast Cancer	Increased expression of miR-10b, miR-155, and miR-195 and decreased miR-34a was associated with disease.	[25]
miR-221	Ovarian Cancer, Melanoma, Lymphoma	Increase expression in several different cancers compared to control serum samples.	[27,28,29]
miR-141, miR-16, miR-92a, miR-92b, miR-103, miR-107, miR-197, miR-34b, miR-328, miR-485-3p, miR-486-5p, miR-574-3p, miR-636, miR-640, miR-766, and miR-885-5p.	Prostate	Levels of these microRNAs were found to be higher in the serum of patients compared to controls.	[21,26]

### 4.2. Circulating MicroRNAs as Prognostic Biomarkers

In addition to their use in diagnosing the presence of cancer, circulating microRNA signatures have also been shown to predict survival in several settings. For example, miR-21, a well characterized oncomiR which functions to enhance invasion and metastasis of the primary tumor across numerous cancer types [13] has been found to be high in the circulation of patients with breast, ovarian, colorectal, gastric, osteosarcoma, and prostate cancer where it predicts for late stage metastatic disease [30,31,32,33,34,35,36,37,38,39]. Likewise, several other well characterized oncomiRs, such as miR-17–92, miR-9, miR-146a, miR-155, miR-181, and miR-221/222 have been found at high levels in the circulation of patients with late stage tumors or metastatic disease (Table 2). Interestingly, several recent studies show that microRNA concentrations are higher in the serum of cancer patients than in healthy controls and higher still in the serum of patients with metastatic tumors, suggesting a progressive rise in serum microRNA levels with disease progression [40,41,42]. Taken together this data suggests that oncomiRs that play a role in driving tumor progression are released from the primary tumor into the circulation where they can be used to gauge patient prognosis.

**Table 2:** MicroRNAs with potential as circulating biomarkers for predicting prognosis of cancer.

Prognostic miR(s)	Disease Setting	Description	Reference(s)
miR-21	Breast Cancer, Ovarian Cancer, Colorectal Cancer, Gastric Cancer, Osteosarcoma, Prostate Cancer	Predicts for late stage and/or metastatic cancer.	[30,31,32,33,34,35,36,37,38,39]
miR-17–92	Breast Cancer, Prostate Cancer, Melanoma, Ovarian Cancer	Circulating levels correlate with metastatic disease.	[41,43,44,45]
miR-9	Melanoma	Serum levels predict distant metastatic lesions.	[46]
miR-146a	melanoma, Gastric Cancer	Plasma and serum levels predict lymph node metastasis.	[34,37]
miR-155	Breast Cancer, Colorectal Cancer, Lung Cancer, Melanoma, DLBCL	Serum or plasma levels associated with metastasis and decreased relapse-free survival.	[41,44,47,48,49]
miR-181	Melanoma	Plasma levels are associated with increased metastasis.	[44]
miR-221/222	Ovarian Cancer, Melanoma, Prostate Cancer, Lymphoma	Plasma/Serum levels associated with disease progression and metastasis.	[27,28,29,38]

### 4.3. MicroRNAs as Biomarkers of Therapeutic Response

A major need in the development of targeted therapies is biomarkers that can be used to monitor patient response. Several recent studies indicate that circulating microRNAs have the potential to be high sensitivity biomarkers for patient response. Indeed, primary tumor expressed microRNAs decrease in the plasma after surgical resection [9]. For example, plasma levels of miR-17-3p and miR-92 decrease after surgical removal of colorectal cancers [50], while miR-184 serum levels decrease after surgical removal of tongue cancer [51]. In breast cancer patients circulating levels of miR-210 were associated with trastuzamab sensitivity, suggesting that this microRNA might be used to monitor response to therapy [52]. Likewise, in addition to miR-155 acting as a diagnostic biomarker to discriminate cancer patients from healthy subjects, it has also been shown to decrease after surgery or chemotherapy treatment in breast cancer patients, suggesting its use as an indicator for treatment response [53]. Taken together these studies indicate that circulating microRNA levels change in response to therapy or surgery (Table 3). However, to date, large scale studies measuring microRNA expression across various treatments and tumor types has not been performed and more detailed studies are needed to determine the utility of specific circulating microRNA profiles in predicting response to specific treatments.

**Table 3:** Circulating microRNAs with potential for predicting therapeutic response.

Therapeutic Response miRs	Disease Setting	Description	Reference(s)
miR-210	Breast Cancer	Associated with trastuzamab sensitivity.	[52]
miR-155	Breast Cancer	Circulating levels decrease after surgery or chemotherapy treatment.	[53]
miR-17-3p and miR-92	Colorectal Cancer	Circulating levels decrease after surgical removal of the tumor.	[50]
miR-184	Tongue Cancer	Circulating levels decrease after surgical removal of the tumor.	[51]

## 5. Circulating MicroRNAs as Mediators of Cell Communication

**Figure 1:**
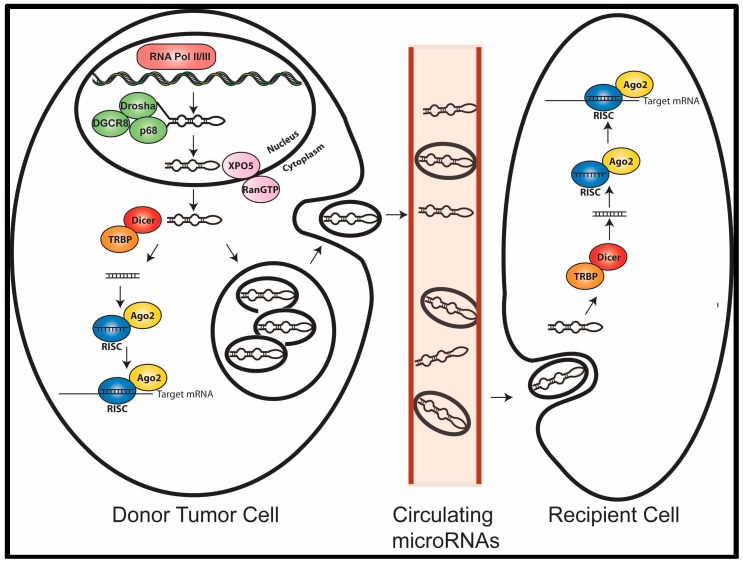
Cell–cell communication through exosomal microRNAs. MicroRNAs contained in exosomes are released from tumor cells where they can enter the bloodstream and circulate through the body to distant sites. These exosomal microRNAs are taken up by recipient cells, where the microRNAs can then suppress target genes in recipient cells.

Given that microRNAs are found in the circulation at much higher levels than any other circulating nucleic acid [10], it is perhaps not surprising that recent evidence points to circulating microRNAs carrying out biological signaling functions by acting as autocrine, paracrine, and endocrine signaling molecules within the body. While Ago-bound microRNAs in the circulation are thought to be non-specific remnants of cell death [54,55], packaging of microRNAs into exosomes is thought to occur through a specialized sorting mechanism [22]. Although, the exact mechanism of sorting remains incompletely understood, exosome profiling has shown that exosomes from different cellular origins contain unique microRNA expression profiles that may reflect the state of the cell of origin [55]. Moreover, tumor cell-secreted exosomes deliver their microRNA cargo to cells in the tumor microenvironment, initiating silencing of mRNAs and reprogramming of the target cell transcriptome, which can lead to the promotion of tumor growth, angiogenesis, immune suppression, and metastasis (Figure 1). Recent work from Melo *et al.* [56] has shown that breast cancer exosomes are specifically enriched in microRNAs compared to normal cells and that these exosomes can instigate non-tumorigenic epithelial cells to form tumors in a microRNA-dependent manner [56]. Additionally, several lines of research indicate that exosomal microRNAs are capable of modulating the tumor microenvironment and eliciting tumor immune responses. For example, exosomal microRNAs secreted by chronic lymphocytic leukemia (CLL) cells have been shown to contribute to reprogramming of surrounding stromal cells, resulting in enhanced proliferation, migration, and secretion of inflammatory cytokines resulting in a tumor-supporting microenvironment [57]. In addition, exosomal miR-9 has been shown to inhibit expression of major histocompatibility complex (MHC) class I preventing recognition of tumor cells by the immune system [58]. Similarly, exosomally secreted miR-21 has been shown to bind to toll-like receptors (TLRs) on immune cells leading to TLR activation and secretion of inflammatory cytokines that promote metastasis [59]. In addition to reprogramming the local tumor microenvironment exosome-derived microRNAs have been shown to function to promote metastasis to distant sites. The miR-200 family has traditionally been characterized as a tumor suppressive microRNA in the cell through its ability to decrease Zeb1 expression leading to the suppression of epithelial-mesenchymal transition (EMT) and metastasis [13]. However, the miR-200 family has been shown to be enriched in the serum of patients with metastatic breast cancer [60] and has recently been shown to confer non-metastatic cells with the ability to colonize distant organ sites of metastasis [61]. Likewise, miR-10b secreted from metastatic breast cancer cells has been shown to confer invasive properties on non-malignant cells [12]. Taken together these findings clearly show that tumor cell-secreted microRNAs contained in exosomes are not simply bystanders of disease, but biologically active molecules that can elicit changes in the tumor microenvironment. This suggests new opportunities for the development of exosomal-based biomarkers and therapies. However, more work is needed to uncover the mechanisms of sorting microRNAs into exosomes and to further define tumor specific exosomal microRNA profiles.

## 6. Conclusions

MicroRNA biology remains an ever-expanding field. Research published over the last decade clearly indicates that microRNAs play a significant role in modulating the signaling pathways that drive cancer progression in the primary tumor. Moreover, some of these microRNAs are released from the tumor into the circulation, where they carry out biological functions and can be utilized as biomarkers. Although it is clear that microRNA profiles can provide unique biomarkers both in primary tumor biopsies and in the circulation, some studies have generated varying results [8], possibly due to different detection methods and difference in sample collection methods. So, further work needs to be done to determine the specificity of microRNA biomarkers for different types of cancer as well as to standardize collection and analysis methodologies. To date, a majority of studies have been performed on relatively small sample sizes, so larger prospective clinical trials are needed to validate these results. Additionally, more work is needed to define the difference between primary tumor and circulating microRNA profiles for individual tumor types. Furthermore, there is a lack of data from comprehensive profiling of circulating microRNA during chemotherapy treatment, which would be useful for identifying new circulating markers of therapeutic response. Ultimately, these findings could form the foundation for developing new biomarkers to improve the clinical course of cancer patients.
